# Efficacy of bumetanide in animal models of ischemic stroke: a systematic review and meta-analysis

**DOI:** 10.18632/aging.205910

**Published:** 2024-06-07

**Authors:** Xiaoyu Sun, Jiadi Hou, Haichun Xu, Huiling Qu

**Affiliations:** 1 Department of Neurology, The General Hospital of Northern Theater Command, Shenyang, China; 2 Department of Psychiatry, Shenyang Jing’an Mental Health Hospital, Shenyang, China

**Keywords:** bumetanide, infarct volume, behavioral recovery, ischemic stroke, meta-analysis

## Abstract

This meta-analysis aimed to describe the efficacy of bumetanide in improving infarct volume, brain edema, and behavioral outcomes in animal models of cerebral ischemia. Embase, PubMed and Web of Science databases were searched from their inception to February 2024 (INPLASY:202430023). Data on the animal species, stroke model, drug dose, time of treatment, method of administration, study quality, and outcomes were extracted and pooled in a meta-analysis. The combined standardized mean difference (SMD) or mean difference (MD) estimates and 95% confidence intervals (CIs) were calculated using random- or fixed-effects models.

Thirteen eligible studies involving >200 animals fulfilled the inclusion criteria and were included in this meta-analysis. Meta-analyses demonstrated that bumetanide treatment significantly reduced cerebral infarct volume (SMD: −0.42; 95% CI: −0.75, −0.09; *p* < 0.01; *n* = 186 animals) and consistently relieved brain edema (SMD: −1.39; 95% CI: −2.06, −0.72; *p* < 0.01; *n* = 64 animals). Subgroup analyses demonstrated that bumetanide treatment reduced infarct volume in transient but not permanent cerebral ischemia models. When administered after the stroke, it was more effective than treatment initiation before the stroke. Eight studies assessed the effect of bumetanide on behavioral function and the results showed that bumetanide treatment significantly improved neurobehavioral deficits (SMD: −2.35; 95% CI: −2.72, −1.97; *p* < 0.01; *n* = 250 animals).

We conclude that bumetanide appears to be effective in reducing infarct volume and brain edema and improving behavioral recovery in animal models of cerebral ischemia. This mechanism needs to be confirmed through further investigation.

## INTRODUCTION

Ischemic stroke, defined as a clinical syndrome of sudden central nervous system dysfunction, seriously threatens human life and imposes a great mental and economic burden on society, families, and individuals [1]. With the increase in the population of older adults in China, stroke-induced disability continues to increase and is the leading cause of death; therefore, developing new stroke treatment methods, including neuroprotective strategies [2]. Although more than 700 drugs have been described for experimental stroke, only tissue-type plasminogen activators have been shown to be effective in human studies [3, 4]. These disadvantages include the risk of bleeding, short effective time window, and unsuitability for many patients [5]. Therefore, it is necessary to investigate other drugs for treating ischemic stroke.

The Na-K-2Cl cotransporter, NKCC1, initially thought to be a small transmembrane protein, is expressed in both immature and diseased central neurons [6–8]. Immunostaining and RNA data indicate that NKCC1 is highly expressed in developing and mature oligodendrocytes (OLs) of the adult mouse brain and is readily detectable in microglia, astrocytes, developing pericytes, and dentate gyrus (DG) progenitor cells [9]. In the hippocampus of adult mice, NKCC1 activity in OLs mediates changes in axonal conduction, which, in turn, modifies synaptic plasticity [10]. It also plays an important role in maintaining electrical neutrality, normal cell volume, and fluid balance in the brain tissues. Moreover, ischemia-induced NKCC1 stimulation plays an important role in stroke pathophysiology, leading to Na+ overload and apoptotic cell death [11]. The diuretic drug bumetanide is a selective inhibitor of NKCC1 that specifically blocks NKCC1 in the brain. Animal experiments have revealed that bumetanide administration can reduce the severity of neuronal and oligodendrocyte damage and create a favorable environment for neurogenesis and axon growth [12–15]. Other studies have shown that bumetanide can reduce cerebral edema and nerve cell injury in rats with focal ischemia-reperfusion injury [16–18]. Several clinical studies have been conducted to evaluate the efficacy of bumetanide for spectrum disorder (ASD), Parkinson’s disease (PD), and neonatal seizures [19–21]. Unfortunately, the results were negative. Moreover, although several studies have suggested that bumetanide may be beneficial in rodents of ischemic stroke, no large clinical trials have explored the effects of bumetanide on stroke, nor have there been systematic reviews and meta-analyses of preclinical stroke literature [22–25]. A systematic review of the effects of bumetanide treatment on ischemic models in an objective and quantitative manner may provide credible and reliable evidence for the use of bumetanide in the clinical treatment of stroke. We systematically assessed the bias in the included studies and summarized the optimal pattern of bumetanide therapy. This meta-analysis may provide significant information for patients with cerebrovascular diseases and reduce disability rates. Our study aimed to systematically determine the efficacy of bumetanide in reducing infarct volume, cerebral edema, and neurological function in rodent models of ischemic stroke and to observe the neuroprotective effects of bumetanide in stroke model and duration subgroups.

## MATERIALS AND METHODS

### Search strategy

We selected relevant studies from publications in PubMed, Embase, and Web of Science from their inception to February 2024. The search terms used were (stroke OR cerebrovascular OR cerebral infarct OR ischemia OR middle cerebral artery OR middle cerebral artery occlusion) AND (rodent OR mouse OR rat) AND (bumetanide OR NKCC1). The language of the publications was limited to English. Two investigators (Jiadi Hou and Haichun Xu) independently retrieved the literature. Disputes that arose during the selection process were discussed and resolved by both parties.

### Inclusion and exclusion criteria and data extraction

The studies included in this meta-analysis and review met the following criteria:

(1) Permanent or transient middle cerebral artery occlusion (pMCAO or tMCAO), ET-1 stroke model, or other focal stroke models were performed on rodents; (2) a control group was set up with a placebo; (3) adequate data on functional outcomes were provided; and (4) experimental studies were presented in the original research articles. The exclusion criteria were as follows: (1) animals treated with bumetanide analogs; (2) neonatal rodents and those using non-rodent models, *ex vivo* and *in vitro* preparations, or humans; (3) hemorrhagic stroke models; (4) not reporting the number of animals in groups; (5) review, editorial, conference abstract, and non-English publications; and (6) repeated publications or duplicate reports and abstracts without full text.

The following details were extracted from each study: (1) document elements: first author and year of publication; (2) experimental animal elements: animal species; (3) MCAO model elements: stroke model and ischemia duration; (4) intervention elements: administration route, dosage, and intervention time point; and (5) experimental outcomes. Studies had to provide data on at least one of the following outcome measures: (1) cerebral infarction volume; (2) brain edema or water content (%); and (3) functional outcomes. The data were presented graphically rather than reported as text and were obtained using a digital ruler software (Universal Desktop Ruler). For each comparison, we extracted the means and standard deviations. All data were independently extracted by two participants, and any discrepancies were resolved by consensus or consultation with a third reviewer (Xiao-Yu Sun).

### Quality assessment

Quality assessment of the studies was performed by two independent reviewers (Jiadi Hou and Haichun Xu). Any discrepancies were resolved through consultation with a third reviewer (Xiao-Yu Sun). We evaluated the methodological quality of the included studies by applying a previously published 11-item quality scale [26], modified as follows: (1) Peer review publication. (2) Strength-response relationship; (3) Random assignment study; (4) Blinded treatment administration; (5) Blind evaluation of results; (6) Physiological parameters monitoring; (7) Sample size calculation; (8) ≥2 outcome parameters were evaluated; (9) Avoidance of anesthetics with marked intrinsic neuroprotective properties (ketamine); (10) Comply with animal welfare regulations; and (11) Statement of potential conflict of interest. Thus, the studies were classified into three quality categories (category I, 8–11 items; category II, 4–7 items; and category III, 0–3 items).

### Statistical analysis

The results of the included trials were analyzed for each endpoint: infarct volume, cerebral edema, and neurological outcomes, including neurological score, memory, and limb function. All analyses were performed using STATA 15.0 software. The I^2^ statistic was used for heterogeneity assessment. When heterogeneity was strong and could not be eliminated, a random-effects model was used instead of a fixed-effects model to obtain the overall MD/SMD and 95% CI [12, 27]. A subgroup analysis was conducted to examine the effect of bumetanide according to the stroke model (permanent or temporary) and time point of intervention (pre-treatment or post-treatment). A leave-one-out sensitivity analysis was performed to evaluate the robustness of the results. Publication bias was checked by a funnel plot, and asymmetry was estimated by Egger’s test and the trim and fill method [28]. Funnel plots were generated to assess the publication bias. If the *p*-value was < 0.01, the significance of all the analyses was accepted.

## RESULTS

### Characteristics of included studies

**Figure 1:**
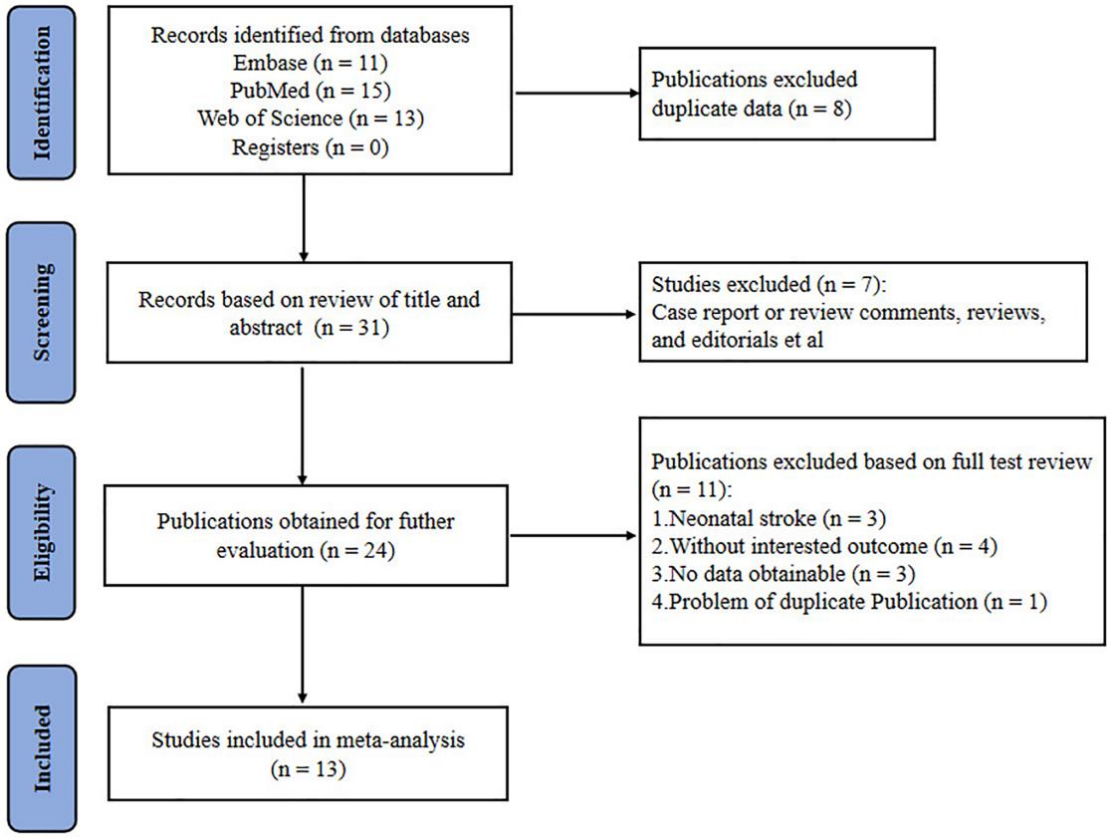
Flow chart for selection of studies.

Screening was performed in two phases: initial screening based on title and abstract, followed by full-text screening of eligible articles for final inclusion. The initial database search identified 39 eligible studies. After removing the duplicates, 31 unique references were identified. The full texts of the remaining 31 records were retrieved for further assessment. Among them, 18 records were excluded because they were reviews, case reports, editorials, inadequate outcomes, neonatal ischemia, and duplicate publications. Finally, 13 studies were included in the meta-analysis (Figure 1 and Table 1) [13–18, 29–35]. Two studies intervened before stroke induction, ten intervened after ischemia induction, and one intervened before and after ischemia. Infarct size was calculated in more than 180 animals, and neurobehavioral deficits were assessed in more than 200 animals. Meta-analysis was performed based on data from 11 studies, and the effects of stroke model and treatment initiation time on infarct volume were analyzed in subgroups.

**Table 1:** Basic characteristics of the included studies.

**Author**	**Year**	**Animal species**	**Stroke model**	**Dose range (mg/kg)**	**Method of administration**	**Control intervention**	**Outcome measures**	**Quality category**
Jun Wang	2022	C57Bl/6J mice	tMCAO (60 min)	10 mg/kg body weight/day	i.p.	DMSO	Infarct volume	II
Fudong Liu	2010	C57BL6 male mice	MCAO (2 hours)	30.4 mg/kg immediately after MCAO and at reperfusion.	i.v.	Vehicle	Infarct volume Neurological deficits	II
Genbao Wang	2014	Adult Wistar male rats	MCAO (2 hours)	30 mg/kg through tail vein 10 min before cerebral ischemia inducing.	i.v.	Saline	Infarct volume brain edema neurological function score	II
Gulnaz Begum	2015	C57Bl/6J male mice	MCAO (60-minutes)	10 mg/kg was injected intraperitoneally at 3, 24 and 48 h Rp, at 3 h reperfusion, and then daily (every 24 h) at the same dose.	i.p.	NKCC1 KO mice	Infarct volume neurological function deficit scoring analysis brain edema	I
Hai Chen	2005	Adult male rats	MCAO (2 hours)	5 to10 mmol/L bumetanide after MCAO	i.p.	NKCC1^−/−^ mice	Infarct volume brain edema	II
Huachen Huang	2019	C57BL/6J mice	t-MCAO for 50 min	10 mg/kg body weight per day was given at 3 hours and the second half dose at 8 hours post-MCAO and twice daily for 1 to 5 days.	i.p.	DMSO	Infarct volume Neurological score	II
Martha E. O’Donnell	2004	Sprague-Dawley rats	MCAO	7.6–30.4 mg/kg in one to four doses of 7.6 mg/kg 20 minutes before initiation of MCAO.	i.v.	Vehicle	Infarct volume brain edema	II
Mohammad Iqbal H Bhuiyan	2017	Adult male Wistar rats	MCAO (2 hours)	The initial dose of 5 mg/kg at 3 h and the second dose of 5 mg/kg at 8-h post-reperfusion were followed by two daily injections	i.p.	Saline	Infarct volume latency times in the turn-alley test Neurological deficit scores	II
Mu XP	2017	Adult male Wistar rats	ET-1 stroke model	0.2 mg/kg/day for 3 weeks on postoperative day 7	micro-injection pump	Saline	Infarct volume Tapered/ledged beam walking test Cylinder test	I
Wang-shu Xu	2016	Adult male Wistar rats	ET-1 stroke model	25 μg/μL of bumetanide was pumped every day, for 21 consecutive days on postoperative day 7	micro-injection pump	Saline	Infarct volume Morris water maze test	II
Wangshu Xu (Mol Neurobiol)	2017	Adult male Wistar rats	ET-1 Stroke Model	0.2 mg/kg/day at 7 days after ischemia	mini-osmotic pumps	Saline	Infarct Volume Beam-Walking Test	II
Yiping Yan	2003	Male rats	MCAO (2 hours)	100 mM bumetanide mi-crodialyzed from 1 h prior to ischemia to 2-h MCA occlusion (pre-ischemic treatment), or during 24-h reperfusion (post-ischemic treatment).	Brain microdialysis procedures	aCSF	Infarct volume brain edema	II
Yiping Yan	2001	Male rats	MCAO (2 hours)	100 mol/L bumetanide solution was made in aCSF throughout the 2-hour MCA occlusion and 24-hour reperfusion.	Brain microdialysis procedures	aCSF	Infarct volume brain edema	II

Abbreviations: MCAO: middle cerebral artery occlusion; ET-1: Endothelin-1; t-MCAO: transient middle cerebral artery occlusion; DMSO: Dimethyl sulfoxide; aCSF: artificial cerebral spinal fluid; IV: intravenous; i.p.: intraperitoneal.

### Effect of the study quality

The results of the quality evaluation are shown in Figure 1. Overall, the median quality (range scale 0–11) of the 13 included studies was moderate (8; interquartile range, 6–8). None of the studies received a score of 0 or were of high quality (9–11). Eleven studies were randomized. Eight studies (61.5%) reported temperature control during surgery, but only four studies (30.8%) reported concealing treatment from the investigator during outcome assessments. In addition, all studies used a blinded treatment with bumetanide.

### Primary outcome assessment: the effect of bumetanide on cerebral infarction volume

**Figure 2:**
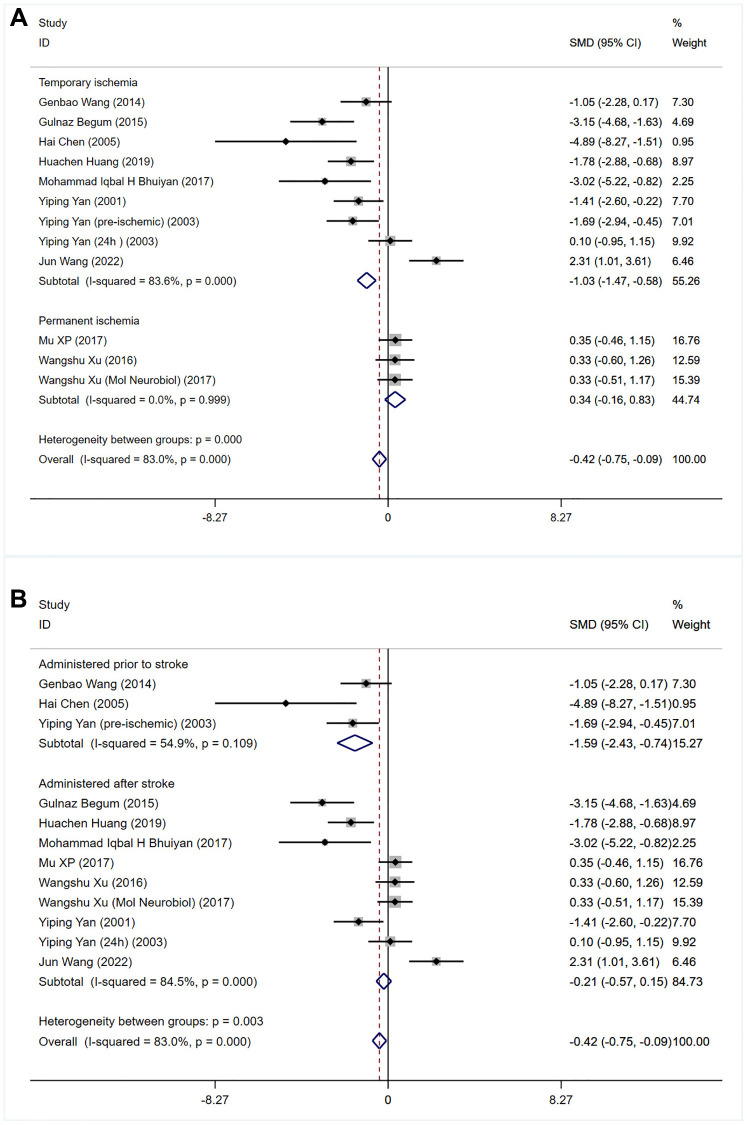
Meta-analysis of infarct volume in rodents treated with bumetanide versus controls was performed, divided by (**A**) duration of ischemia (permanent vs. transient) and (**B**) timing of treatment before or after the onset of ischemia.

The effect of bumetanide upregulation on cerebral infarction volume was assessed in eleven studies (Table 1) [13–16, 18, 29–33, 35]. These studies measured the cerebral infarction volume by histological assessment of excised brain (tetrazolium chloride (TTC), cresyl violet) and were recorded in mm^3^. Meta-analysis indicated that compared with the effects of a vehicle, bumetanide significantly reduced cerebral infarction volume (SMD for overall effect: −0.42; 95% CI: −0.75 to −0.09; *p* = 0.000; I^2^ = 83%; Figure 2). Subgroup analysis showed that bumetanide reduced infarct volume in transient, but not permanent ischemic models (SMD: −1.03; 95% CI: −1.47 to −0.58; *p* = 0.000; I^2^ = 83.6% and SMD:0.34, 95% CI: −0.16 to 0.83; *p* = 0.999; I^2^ = 0%, *p* = 0.999 respectively; Figure 2A). Bumetanide administration after ischemia induction significantly reduced the volume of cerebral infarction relative to administration before ischemia (SMD: −0.21; 95% CI: −0.57 to 0.15; *p* = 0.000; I^2^ = 84.5% and SMD: −1.59, 95% CI: −2.43 to 0.74; *p* = 0.109; I^2^ = 54.9%, respectively; Figure 2B). Sensitivity analyses showed that the overall findings did not substantially reverse after removing any one trial, indicating that the results were reliable.

### Secondary outcome assessment: the effect of bumetanide on brain edema

**Figure 3:**
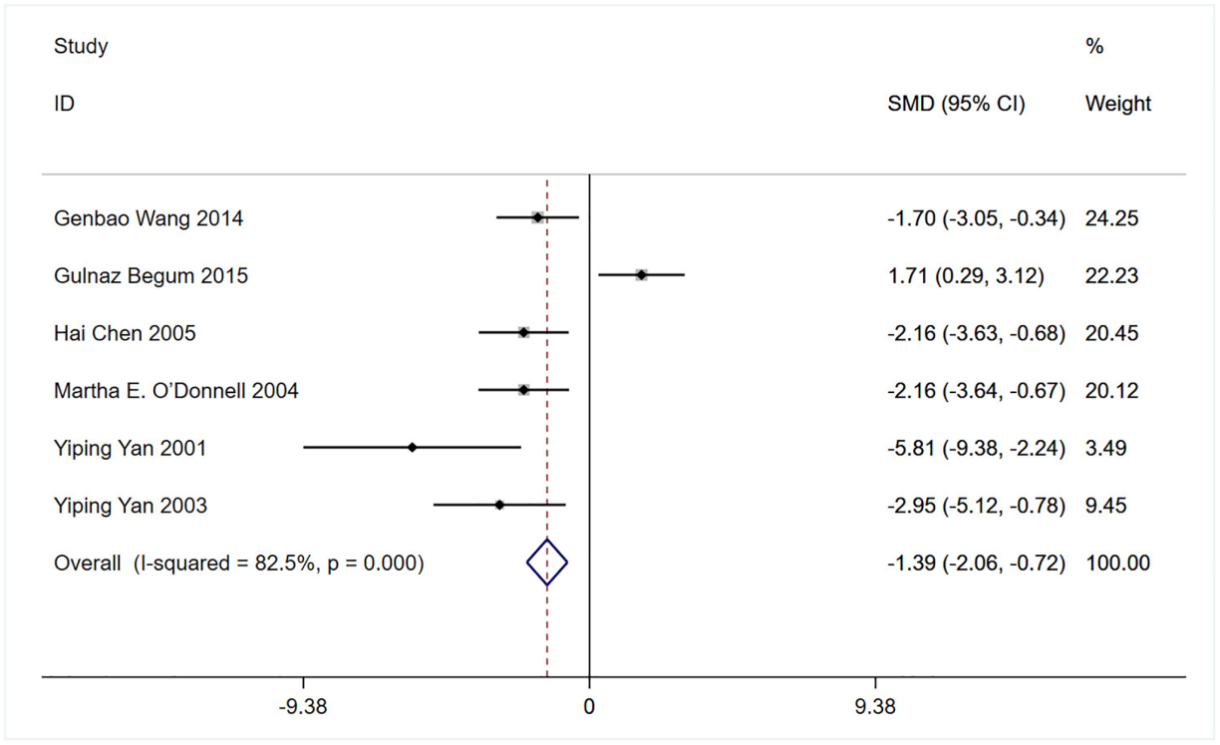
Meta-analysis comparing brain edema (Water content (%)) in rodents receiving bumetanide intervention compared to controls.

Six of the 13 eligible studies, which included 64 rodents, assessed the effects of bumetanide treatment on brain edema (water content (%)) [16–18, 32, 33, 35]. A meta-analysis of findings demonstrated that brain edema was significantly lower in animals treated with bumetanide than in controls (SMD: −1.39; 95% CI: −2.06 to −0.72; *p* = 0.000; I^2^ = 82.5%; Figure 3).

### Secondary outcome assessment: the effect of bumetanide on neurological function

**Figure 4:**
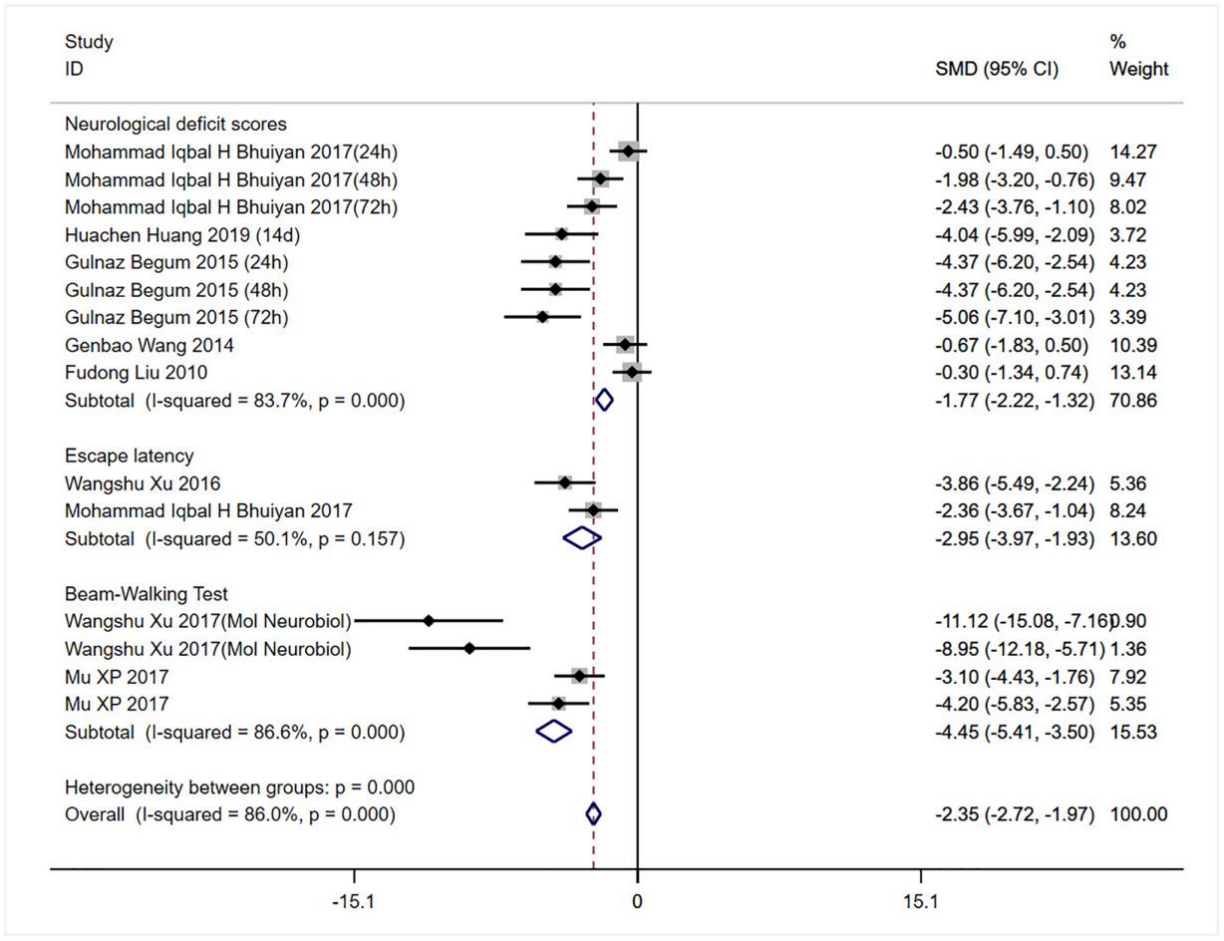
Meta-analysis comparing neurobehavioral function indicated by neurological score, Morris water maze test and tapered/ledged beam walking test in rodents receiving bumetanide intervention compared to controls.

Eight studies assessed the effects of bumetanide on the neurological function in stroke models (Figure 4) [13–15, 30–34]. Two studies repeatedly assessed the neurological function during the follow-up period. Overall, bumetanide treatment was shown to have a protective effect by improving neurological function compared with the control group (SMD: −2.35; 95% CI: −2.72 to −1.97; *p* = 0.000; I^2^ = 86%). Subgroup analyses suggested that bumetanide treatment improved neurological deficit scores and limb function compared to the control group (SMD: −1.77; 95% CI: −2.22 to −1.32; *p* = 0.000; I^2^ = 83.7%; SMD: −4.45, 95% CI: −5.41 to −3.50; *p* = 0.000; I^2^ = 86.6%, respectively; Figure 4), but not improved cognitive function (SMD: −2.95; 95% CI: −3.97 to −1.93; *p* = 0.157; I^2^ = 50.1%). Moreover, the different species did not affect the improvement of neurological function (SMD: −1.77, 95% CI: −2.22 to −1.32; *p* = 0.000; I^2^ = 83.7%; Supplementary Figure 1). Sensitivity analyses showed that the exclusion of other studies did not affect the results of the overall analysis.

### Publication bias

**Figure 5:**
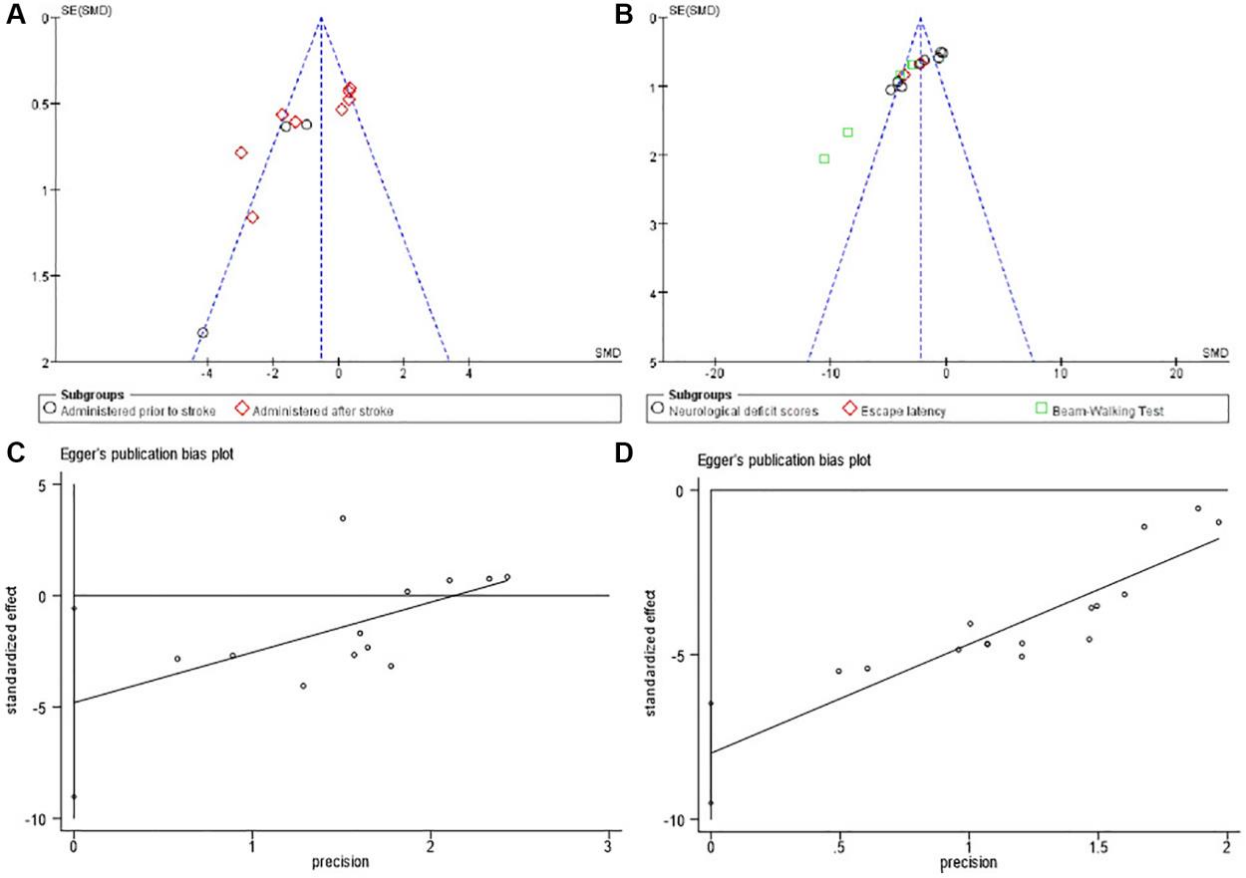
Bias assessment plot for the effect of bumetanide on infarct volumes and neurological function by funnel blot (**A**, **B**) and Egger’s test (**C**, **D**).

Finally, we sought to identify whether the effect of small studies may contribute to publication bias in bumetanide analysis. Funnel plot (Figure 5) evidenced asymmetry, indicating a potential publication bias. Through Egger’s test, the publication bias existed in infarction volume and neurological function data (*p* = 0.030, *p* = 0.000 respectively). Therefore, trim and fill analysis was further used to estimate the risk of publication bias on the results, but no significant bias was detected.

## DISCUSSION

Our systematic review and meta-analysis highlight the value of bumetanide in the treatment of cerebral ischemia, with evidence that it can significantly reduce infarct size and cerebral edema and improve neurological function. Subgroup analysis suggested that a transient ischemic model and post-stroke pharmacotherapy may be more effective. Funnel plots showed low published bias for the main outcomes (cerebral infarction volume and neurological function); however, this was difficult to evaluate objectively because relatively few studies were included [36]. Finally, infarct volume was the primary outcome indicator in this meta-analysis, as it is the most widely reported outcome indicator associated with stroke severity in clinical studies and has translational potential.

Clinical investigations have suggested that infarct size is associated with stroke severity [37]. Therefore, reducing infarct size has the potential to treat ischemic stroke. The data from the present review suggest that bumetanide promotes the recovery of infarct size. However, the possibility that bumetanide reduced infarct volume cannot be entirely attributed to reduced edema. For example, bumetanide may promote neurogenesis, strengthen existing neuronal networks, and modulate signaling. The exact mechanisms underlying the beneficial effects of bumetanide need to be fully explored in further studies to provide a new therapeutic target for reducing neurological damage that occurs after stroke. The timing of bumetanide administration is another important factor that needs to be considered. Our meta-analysis showed that bumetanide uses after the acute phase of stroke reduced the size of the cerebral infarction. However, Wang et al. showed bumetanide treatment was most effective in alleviating behavioral changes when administered 10 min before cerebral ischemia induction, and this dosing period had a larger effect size [33]. Whereas this is very difficult to transform it clinically because bumetanide cannot be administered in advance without cerebral infarction. Our meta-analysis also showed that the transient focal cerebral ischemic stroke model may exert higher efficacy because it closely mimics ischemic stroke [38]. In addition, we defined the animal model and outcome indicators before the meta-analysis, which reduced bias in the selection of the included studies. However, while 50% or more of the analyzed studies were produced in the laboratory of Dandan Sun in Pittsburgh, whose reputation is outstanding, the potential for bias was also enhanced.

Cerebral edema after cerebral ischemia and hypoxia is a basic pathophysiological process and a common cause of aggravation, deterioration, and even death, with the incidence of ischemic cerebral edema accounting for 10%. It is defined as an increase in brain tissue volume due to increased fluid content in the brain tissue, which can be life-threatening [38]. Recent studies have shown that bumetanide can reduce the extent of brain necrosis, neurological function scores, and the degree of brain edema in rats after treatment. Similarly, our meta-analysis revealed that bumetanide administration reduced ischemia-induced brain edema. However, the mechanisms underlying cerebral edema remain unclear. Ion channels, transporters, and exchange proteins may be involved in the development of cerebral edema.

In addition to infarct volume, behavioral outcomes need to be assessed because they have the greatest clinical relevance [39]. Until now, it has been argued that bumetanide can alter behavioral recovery in patients with stroke. However, our meta-analysis showed that bumetanide improved neurological deficits, including limb motor function as assessed by the beam-walking test, nevertheless no effect on cognitive function as assessed by escape latency. In addition, the different species did not affect the improvement of the neurological function.

Although our systematic review and meta-analysis validated the ability of bumetanide to reduce infarct size and improve neurological function after ischemic stroke, the underlying mechanisms are poorly understood. Studies in rodent models have shown that bumetanide can promote neurogenesis and axon germination in stroke rats, thereby improving sensorimotor recovery, suggesting that the behavioral effects of bumetanide are related to neuroplasticity [14, 15]. Moreover, mice treated with reduced-subunit-containing GABAARs or low doses of GABAA antagonists have been reported to achieve better behavioral performance, suggesting that reducing excessive GABA-mediated tonics may benefit functional recovery after stroke [40, 41]. As mentioned above, bumetanide facilitates and enhances functional recovery after stroke through NKCC1-mediated GABAergic signaling in the acute phase. WNK3 is an upstream regulator of NKCC1 and activates NKCC1 via phosphorylation [32, 42]. Recent studies have shown that nerve injury can be alleviated, and neurological function recovery can be accelerated by reducing NKCC1 stimulation sites and downregulating NKCC1 expression on wNK3-induced cell surfaces [31, 32]. Therefore, bumetanide treatment had an effect similar to that of GABAA receptor antagonists and WNK3 knockout mice, which may promote functional recovery after stroke via neuroprotection [43].

### Limitations

There are several limitations to drawing definitive conclusions. First, our study included only data published in English, which may have led to a certain degree of selection bias. Second, these findings must be interpreted with caution because of the small number of studies included, especially in the subgroup analyses. In addition, other indicators such as blood-brain barrier damage and cerebral perfusion were not evaluated due to the limited data. Third, we judged that the methodological differences in administration methods and doses were too great to justify the meta-analysis; therefore, the optimal dose for stroke treatment was unclear. Fourth, the heterogeneity between the included studies could not be evidently decreased, even if subgroup and sensitivity analyses were performed. Fifth, although all studies used blinding treatment with bumetanide, only four studies concealed treatment from the investigator during outcome assessments; therefore, there is a chance of overestimation of the results. Finally, we performed data extraction from the graphics using a digital ruler software, which may have altered the original data and affected the results.

## CONCLUSIONS

The advantage of this study is that it is the first meta-analysis of the efficacy of bumetanide in the treatment of ischemic stroke in an animal model. In conclusion, our meta-analysis confirmed the effectiveness of bumetanide therapy in reducing infarct size and improving functional recovery after stroke. Subgroup analysis showed that transient ischemia may be more effective than permanent ischemia and that post-stroke therapy may be more effective than advanced treatment. Altogether, our findings may help to design future experimental and clinical studies.

## Supplementary Materials

Supplementary Figure 1
