# Supplementary material for: Efficacy of bumetanide in animal models of ischemic stroke: a systematic review and meta-analysis
Source: Aging (Albany NY). 2024 Jun 7;16(11):9959–71. doi: 10.18632/aging.205910 (PMC11210250; doi:10.18632/aging.205910)
Supplement: Supplementary Figure 1 [file aging-16-205910-s001.pdf]

SUPPLEMENTARY FIGURE

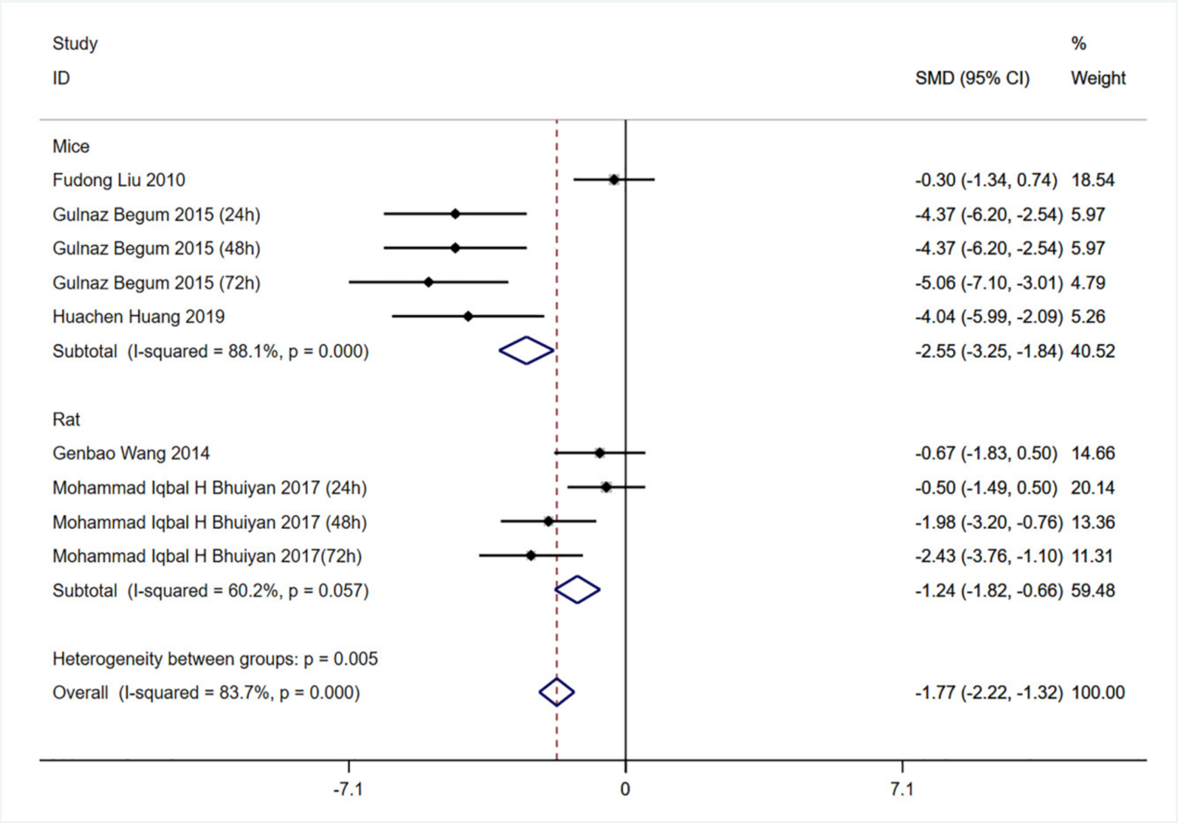

Supplementary Figure 1. Forest plot shows the effects of different species on neurological score.
